# Design of a Hybrid 3D-Printed Composite Material Based on Non-Woven Needle-Punched Fabrics with Radio-Absorbing Properties

**DOI:** 10.3390/polym17172324

**Published:** 2025-08-27

**Authors:** Victor Nazarov, Fedor Doronin, Alexander Dedov, Andrey Evdokimov, Georgy Rytikov, Mikhail Savel’ev

**Affiliations:** Faculty of Printing Industry, Moscow Polytechnic University, 107023 Moscow, Russia

**Keywords:** filament, additive manufacturing, carbon fiber, mathematical modeling, polymer substrate, surface modification, hybrid material, non-woven, composite

## Abstract

The paper proposes a manufacturing technology for the non-woven/3D-printed (N3DP) hybrid material (HM) with improved radio-absorbing properties. We have fabricated the needle-punched non-woven felt and impregnated it with the carbon fibers containing UV-curable photopolymer resin. The functional 3D-printed layer was attached to the highly porous, deformable polymer substrate by the fused deposition modeling (FDM) technique. The preliminary bulk modification of the filament was realized with the IR- and UV-pigment microcapsules filling. The combination of additive prototyping and non-woven needle-punched fabrics surface modification (by the electrically conductive elements 2D-periodic system applying) expands the frequency range of the electromagnetic radiation effective absorption. It provides the possibility of a reversible change in the color characteristics of the hybrid material surface under the influence of the UV and IR radiation.

## 1. Introduction

The radio engineering and electronic devices are applied in different consumption and production fields. The human-made electromagnetic radiation of a wide frequency range (from 3 kHz to 3000 GHz) is observed in a single functional and constructive space of buildings, structures, and environment. The increasing number and the complexity of used electro-technical components lead to a regular growth of the generated electromagnetic field intensity [1,2,3,4].

It is necessary to provide the electromagnetic compatibility (EMC) for the correct electronic equipment joint operation [5,6]. This task is often solved by the radio-absorbing materials using [7,8,9]. However, the problem of obtaining wide-band electromagnetic radiation absorbing non-woven material (with a certain weight and thickness) has not been completely solved [10,11,12].

A combination of the composite materials’ rational choice and the technologies for various conductive elements’ 2D-periodic systems forming (on the “shielding” product’s surface) perspective. They can be used to expand the frequency range of the effective electromagnetic radiation absorbing [13,14,15].

Another fundamental–applied task of modern materials science (relevant for a number of high-tech applications, including printed electronics and sensors) is the design and manufacture of the “intelligent” [16,17,18,19] hybrid polymer systems. Also, they can contain the thermo- and photochromics [20,21,22,23] with the adjustable optical properties [24,25,26].

The current implementation of the additive prototyping makes it possible to demonstrate the economic and technical advantages of the 3D production processes over the traditional ones. It also provides the opportunity for digital design and manufacture of new polymer materials and products’ prototypes with macroscopically ordered structure [27,28,29].

One of the most common polymer products’ additive manufacturing techniques is the free form fabrication (FFF) technology [30,31,32]. It allows combining and using simultaneously the achievements and the advantages of both classical materials science methods and modern computer-aided design (CAD) [33,34].

FDM technology is the process of 3D-product forming (usually its shape is difficult to manufacture with other techniques) as a result of layer-by-layer deposition of a filament [35,36]. The last is extruded through a die (with a diameter of 100–400 microns) of a 3D-printer’s “printing head” that moves according to an algorithm specified by a digital model [37,38]. Various possibilities of this technology used in telecommunications, microelectronics, aerospace, machine-building industries, construction, and medicine (microfluidics, sensors, and biotechnologies equipment) are described in detail [39,40,41].

The most common thermoplastic materials for FDM-3D printing are a copolymer of acrylonitrile, butadiene, and styrene (ABS), polylactide (PLA), and polyethylene terephthalate glycol (PETG) [42,43,44]. The latter is characterized by sufficiently high chemical resistance, mechanical strength, optical transparency, etc. [45,46,47]. The PETG is often used for implants, medical equipment components, and pharmaceutical packaging due to its ability to withstand sterilization procedures with abrasive materials [44,45,47]. The advantages of PETG as a functional additive prototyping material include high interlayer adhesion, low shrinkage, and a low level of the ingredients’ desorption [44,45,47].

We have proposed a technology for manufacturing the hybrid materials (HM) based on the non-woven synthetic fibrous needle-punched felt (NWF) impregnated with carbon fibers containing UV-curable resin. The composite filaments’ layers (containing microcapsules [48] of thermo- and photochromic pigments) have been attached onto the surface of NWF by the 3D-extrusion prototyping technique. The HMs’ radio-absorbing properties have been studied.

## 2. Materials and Methods

The created radio-absorbing products’ prototypes were made from a hybrid material based on the non-woven felt (NWF) with a 3D-printed layer of a bulk modified polyethylene terephthalate glycol (PETG) filament. The polyethyleneterephthalate (PET) fibers with a linear density of 0.33 tex (20–22 microns in diameter) were used to manufacture the NWF. The applied NWF surface reinforcing additive layer was formed on the basis of PETG (Shijiazhuang Tuya Technology Co., Ltd., Shijiazhuang, China) bulk modified with the photo- and thermochromics containing microcapsules (Hali Ind., Changzhou, China). All variants of the composite filaments were formed in a modernized twin-screw extruder (Moscow Polytech, Mashplast, Moscow, Russia) at a temperature of 240 °C in accordance with [49].

### 2.1. The Technology of Radio-Absorbing Hybrid Material Manufacturing

This section describes the technology of the prototypes’ manufacturing and the approaches to studying their properties.

#### 2.1.1. The Main Stages of the Technology

**Figure 1:**
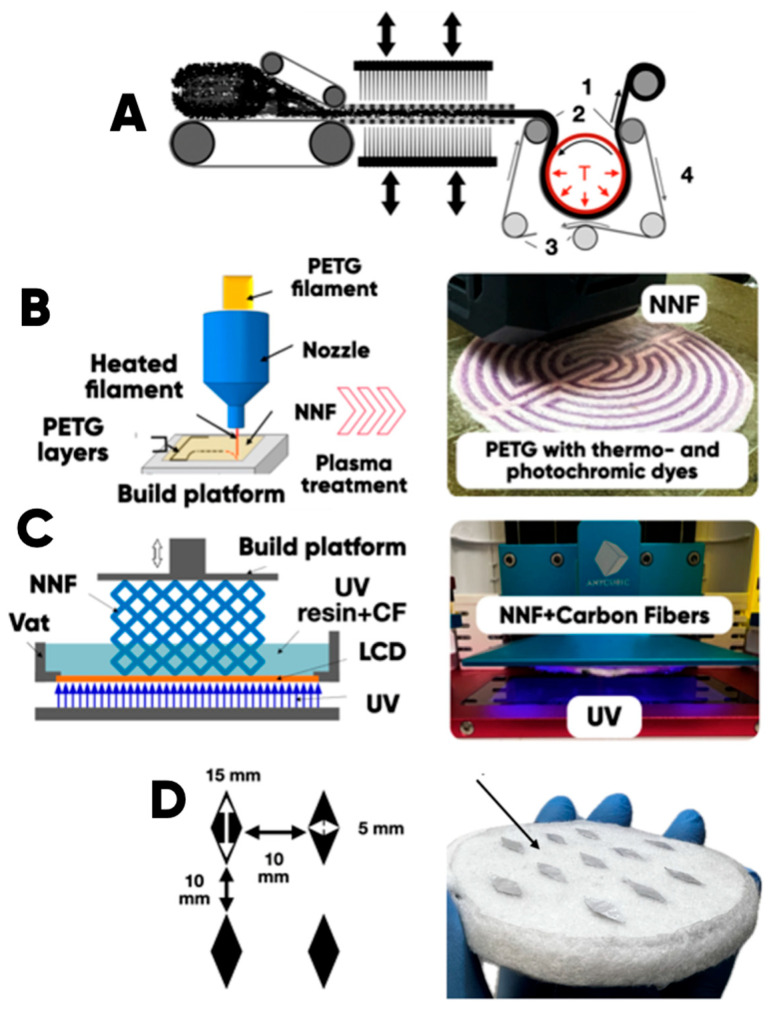
The HM-manufacturing technology stages: (**A**) forming the non-woven felt (NWF) Adapted from [50,51] MDPI, 2024; (**B**) functional PETG-layers 3D printing; (**C**) the impregnation of the NWF with carbon fibers containing UV-photo-curable resin; (**D**) the aluminum foil rhombs 2D-periodic system. The explanation of (**A**): 1—modified material; 2—heated shaft; 3—guide shafts; 4—conveyor belt. A thin arrow shows the direction of movement of the conveyor belt, and a thickened arrow shows the movement of the fabric. The 3D printed on the NWF-surface functional PETG layers (**B**) contain thermo- and photochromic microcapsules. (**C**) decoding: UV—ultra-violet radiation; UV resin + CF—carbon fibers bulk-modified UV-photo-curable resin.

**Figure 2:**
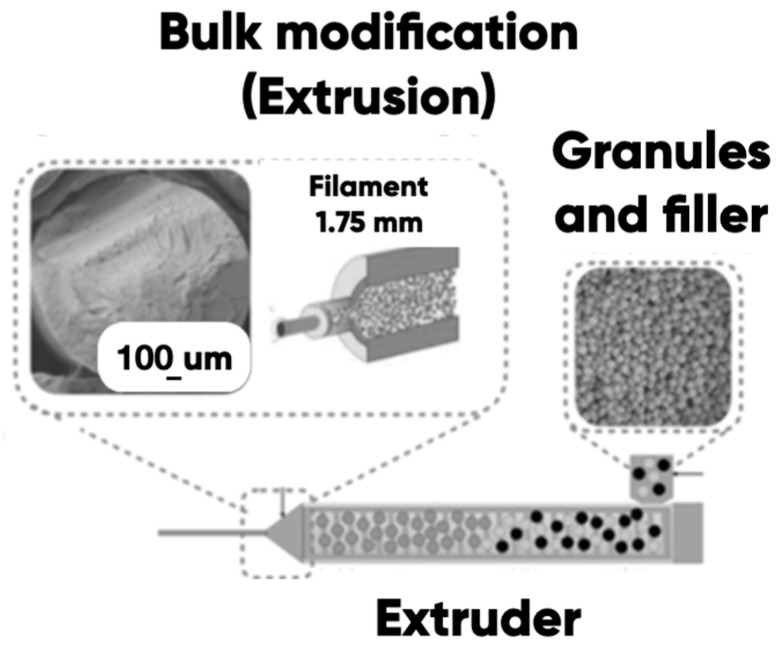
The thermo- and photochromic microcapsules filled the PETG-filament creation Adapted from [52,53], Elsevier, 2021 and AccScience Publishing, 2022.

**Figure 3:**
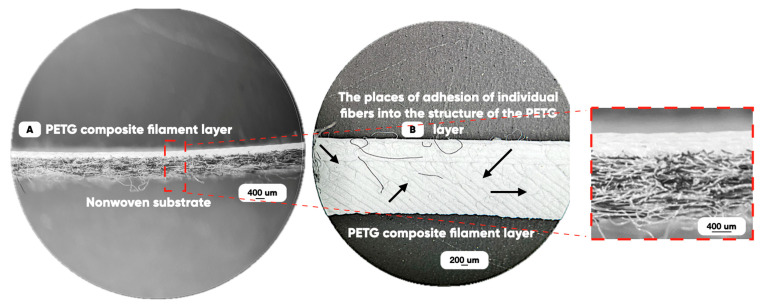
The profile (**A**) and the frontal (**B**) optical images of the NWF/PETG-hybrid material.

**Figure 4:**
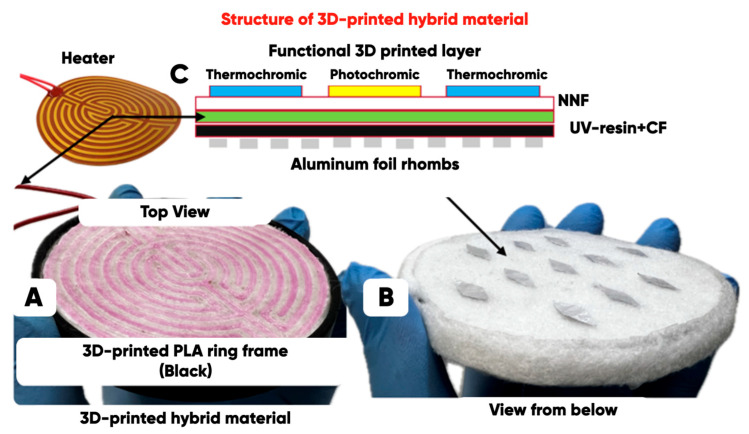
The type (**A**,**B**) and the structure (**C**) of a hybrid material component.

The main stages of a hybrid material (with a containing thermo- and photochromic microcapsules functional composite layer) production (Figure 1) are as follows:

(1) The formation of NWF-base (determining mechanical properties of the hybrid material for radio-absorbing products manufacturing) by thermomechanical treatment of the polymer fibers (Figure 1A);
(2) The FDM-prototyping of the 3D-printed pattern (simulating the heating element design) on the surface of the NWF-base (Figure 1B);
(3) The creation of a functional radio-absorbing layer of the carbon fibers (CFP-7-100, Yataida High Tech Co., Ltd., Shenzhen, China), bulk-modified UV-curable resin (ABS-live Resin Pro 2, Anycubic, Shenzhen, China) in the NWF-based near-surface layers with the photopolymerization 3D-printing technique (Figure 1C);
(4) Applying the 2D-periodic system of aluminum foil rhombs onto the NWF-base surface provides an extension of the effective radio absorption range (Figure 1D).

#### 2.1.2. The Hybrid Material NWF Basis

The examples of the hybrid material NWF-basis manufacturing were carried out by roller calendaring (thermomechanical treatment). We used the PET fibers with a linear density of 0.33 tex (20–22 microns in diameter) at different temperatures (T ∈ [130; 210], ∆T = 20 °C) and speeds (v ∈ [1.5; 11.5], ∆v = 2 m/min) (Figure 2) The felt was produced by mechanical molding at the Schninbau (Nomaco GmbH & Co, Eberbach, Germany) industrial plant in accordance with [50]. The canvas was 1.0–1.2 mm thick and weighed 0.10–0.15 kg/m^2^. The hardening of the fibrous canvas was performed on a needle-punched Dilo unit (Di loDi-Loom (Eberbach, Germany)). The density of the main piercing of the fibrous felt was 180 cm^−2^.

#### 2.1.3. The PETG-Based Composite Filament

We have performed a bulk modification of PETG with photo- and thermochromics containing microcapsules (Hali Ind., Changzhou, China). The extrusion of the composite filament was carried out at a temperature of 240 °C (Figure 2). The single-screw extruder (Filastruder, Twinsburg, OH, USA), the EPS 20 × 25 extrusion line (Polymermashservice, Kuznetsk, Russia), and the modernized twin-screw extruder (Moscow Polytech, Mashplast, Russia) were used. Their nozzles’ diameters were from 100 to 600 microns.

We have produced a prototype at the required quality rate (without low interlayer adhesion, voids, non-prints, etc.) with 1 vol.% of IR and UV pigments containing microcapsules in the PETG-matrix. The functional patterns formation on the NWF surface was carried out using FDM technology by the Anycubic Kobra Go 3D printer (Anycubic, China). Its print-head die diameter was 400 microns; the extrusion temperature was 240 °C; and the 3D-printing speed range was 10–40 mm/min) (Figure 1B). The profile and the frontal optical images of the NWF-basis surface with UV- and IR-pigments bulk-modified PETG-filament layers are shown in Figure 3.

#### 2.1.4. The Aluminum Foil 2D-Periodic Pattern

The application of a 10-micron-thick 2D-periodic aluminum rhomb structure to the NWF surface with a high adhesive glue layer [10,11,12] (Figure 1D) made it possible to expand the effective radio absorption frequency range. It is important for the development of the materials necessary to increase the electromagnetic compatibility of the medical, scientific, construction, etc., radio engineering, and electronic devices located in an enclosed space.

### 2.2. The Techniques of the Hybrid Material Components Structure and Properties Studying

The physics–mechanical properties tests of the NWF (manufactured at different rolling temperatures) and NWF/PETG hybrids (formed by the FDM-3D printing) were determined using a Zwick Roell BZ1.0 universal bursting machine (ZwickRoell Group, Kennesaw, GA, USA).

We have investigated the structure and the chemical composition (mapping the planar distribution of chemical elements) of the composite filaments. It was carried out using a JSM-7500 FA auto emission scanning electron microscope (JEOL, Akishima-sh, Japan) and an Oxford X max 80 detector with a SATW window. There were used the accelerating voltage of ~10 kV, the electron current of ~1 nA, and the average analysis depth of ~0.4 microns.

A standard (“horn”) technique was used to measure the coefficient of the electromagnetic reflection (EMR) from the samples’ surface [10,11,12]. The reflection coefficient was determined using a panoramic meter with the pyramidal horn antennas. The equipment allows us to measure the reflection coefficient in the frequency range from 2 to 34 GHz. The antenna’s output aperture for 2 to 12 GHz EMR measurements is a square with 180 mm × 180 mm sides (for 12 to 34 GHz—120 × 120 mm). The horn antenna has an opening angle of no more than 12° to provide the free space alignment and to create a sufficiently flat electromagnetic wave front. The sample was placed on a metal plate, which provided a complete reflection of passed through the material electromagnetic radiation. The reflection coefficient was calculated using the $R=10\cdot lg\left(P_{ref}/P_{inc}\right),$ where $P_{ref}$ and $P_{inc}$ are the reflected and the incident radiation powers.

## 3. Results and Discussion

The structure of the items (made from the developed hybrid material with an extended range of radio-frequency electromagnetic radiation absorption) is shown in Figure 4. The color characteristics direct regulation (under the influence of UV and/or IR radiation or heating) is also available for them.

The pattern of 3D-printed IR and UV pigments containing microcapsules bulk-modified PETG is represented in Figure 4A; the non-woven needle-punched felt with aluminum rhombs system is in Figure 4B.

### 3.1. The Mechanical Properties of the Prototypes

**Figure 5:**
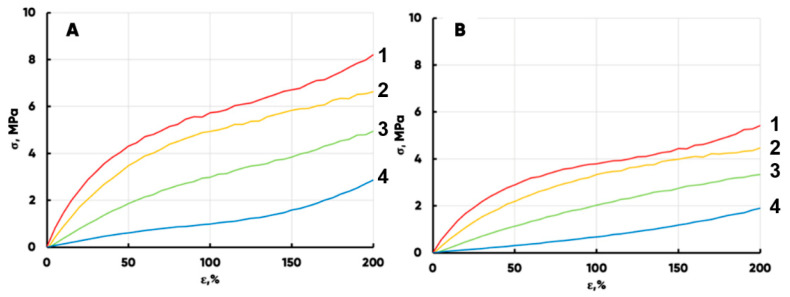
The dependences of the conditional stress on the relative elongation in the transverse (**A**) and longitudinal (**B**) directions for the initial felt (1) and for the variations obtained on its basis at a processing speed of 1.5 m/min and a calendering temperature of 150 (2), 190 (3), and 210 (4) °C.

It is widely known that a technologically determined anisotropy of the structure and, as a result, the properties of the manufactured non-woven felts are observed [54,55,56]. The classical technique for determining the polymer materials’ physics–mechanical properties is to measure the conditional stress and elongation of a sample using bursting machines [57,58,59]. Figure 5 shows the obtained dependences of the mechanical stress (MPa) on the relative elongation (%) of the samples. There are ones for the initial (1) and the thermomechanical treated (150 (2), 190 (3), and 210 (4) °C) at 1.5 m/min speed samples.

The NWFs’ physics–mechanical properties anisotropy is preserved in all cases (Figure 5). At the same time, it is possible to simulate the “longitudinal” piezo-deformation properties of the 150 °C-treated felt with the “transverse” deformation curve of the 210 °C calendered one. The “green” curve (3) in Figure 5A practically coincided with the “red” curve (1) in Figure 5B. Thus, special attention should be paid to the correct choice of the coordinate system axes’ mutual orientation when industrial scaling of the developed technology. This is due to the anisotropic structure of the NWF basis and is necessary to provide the required level of mechanical strength for the hybrid material-made products.

**Figure 6:**
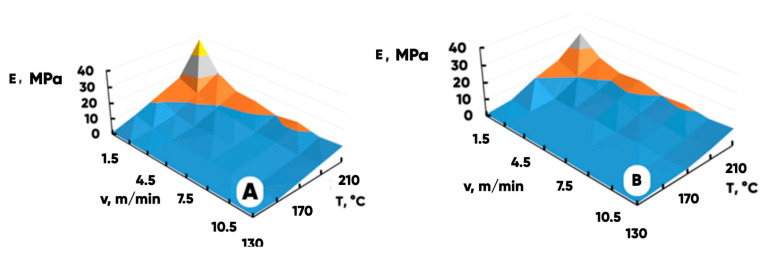
The average values of the effective strength modules E(MPa) calculated for the NWF basis obtained at different temperatures and speeds of the thermomechanical processing: the samples were stretched in the transverse (**A**) and the longitudinal (**B**) directions.

Another important characteristic of the NWF-basis structural properties is the effective strength modulus E(MPa) of a highly porous material (it is to some extent correlated with Young’s modulus for an equivalent model quasi-continuous medium) [60,61,62]. Figure 6 shows the dependence of the NWF-basis effective longitudinal and transverse strength modules on the temperature, T(°C), and the speed, v(m/min), of calendaring.

An exponential function was proposed to model the corresponding dependencies:

(1) $$E=A\cdot exp\left(\frac{\left(T-T_{0}\right)}{\delta T}-\frac{v}{\delta v}\right)$$

Here, E is the effective NWFs’ strength modulus. T, v are the temperature and the calendering ratio. A is the range of E-variation. $T_{0}$ is the calculated value of the temperature (in Kelvin degrees) at which the NWF is expected to collapse during the calendaring. δT is the temperature range within E varies by e times. δv is the rolling speed changing interval providing a decrease in E by e times.

**Figure 7:**
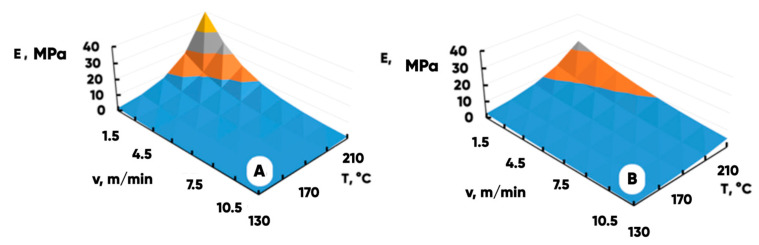
The results of the transverse (**A**) and the longitudinal (**B**) NWFs effective strength modules dependences’ approximation by the model function (1).

The values calculated using model (1) are shown in Figure 7A,B. The quality of data approximation with the model and the results of its parameter specification are presented in Table 1.

The quality of the experimental data with the model function approximation was quantified by the determination coefficient [63].

Calculating:

(2) $$R^{2}=1-\frac{\sum_{n=1}^{N}{\left(E_{n}^{e}-E_{n}^{t}\right)}^{2}}{\sum_{n=1}^{N}{\left(E_{n}^{e}-\bar{E}\right)}^{2}}$$

Here, the ${\left\{E_{n}^{e}\right\}}_{N}$ and ${\left\{E_{n}^{e}\right\}}_{N}$ are, respectively, the sets of measurement results and calculations according to (1). $\bar{E}$ is the average value of the effective modulus of strength for all the experimental samples.

The results of model (1) parameter specification by the simplex method and the data approximation quality (2) are presented in Table 1.

The technological task of achieving the concrete NWFs’ effective strength modulus can be solved by choosing a number of materials’ processing speed and temperature combinations [64,65,66].

It is indicated by the constant level line on the 3D graph of the *E(v; T)* dependence. The ambiguity in the formal solution of the same target achieved by several factors changing should be interpreted as a possibility to regulate at least one more property of the non-woven felt than the effective strength modulus.

Adding a layer of bulk modified PETG-filament to the NWF surface significantly improves the physics–mechanical properties of the hybrid material-made products too [67,68]. The tensile strength of the NWF/PETG hybrid (constructed on various rolling temperatures NWF basis) is 2–5 times higher than the value of this characteristic for the initial material. It is due to the implantation of many individual fibers of the NWF basis into the PETG layer during the curing (Figure 3).

The results of the manufacturing at different temperatures and the same calendering speed (6 m/min), NWF and corresponding NWF/PETG-hybrid material samples’ ultimate mechanical strength investigation are presented in Table 2.

The features of the FDM additive prototyping over the NWF substrate (Figure 3) make it possible to adjust the strength of the adhesive interaction [69] between the non-woven felt and the cured filament by choosing some parameters of the “3D rasterization” (slicing [38,70,71]). They are the relative location of the filaments’ “strokes” and the fibers’ common orientation directions; the distance between adjacent layers; the melt application speed, etc. However, it is necessary to modify the 3D-printer standard settings that significantly affect the structure and the properties of the manufactured items.

### 3.2. The Results of NWFs’ Structure and Optical Properties Determination

**Figure 8:**
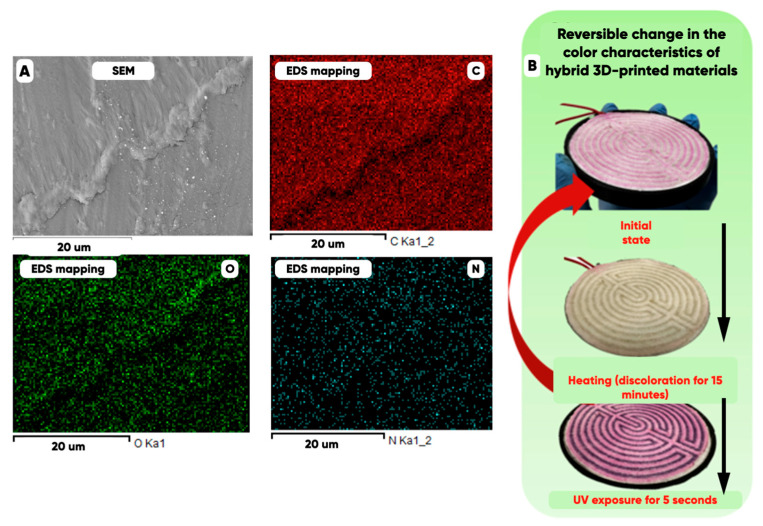
The SEM image and the distribution of carbon (C), oxygen (O), and nitrogen (N) over the surface of a PETG filament containing 1 mass.% of thermochromic microcapsules (**A**). The reversible change in the hybrid material color characteristics depending on the IR- (“heating”) and UV-radiation effect (**B**).

It is known that the color [72,73] of the rough surface objects is an integral spectral characteristic of the optical-frequency range electromagnetic radiation’s diffusive reflection (taking into account the physiological features of human vision [74]). So it was necessary to provide the uniformity of thermo- and photochromic microcapsules distribution over the filament polymer matrix volume. It offers the direct control of the NWF/PETG-hybrid functional layer’s color characteristics by the IR- and/or UV-radiation influence [75,76]. The rational choice of the filament formation rate, the temperature spatial distribution in the extrusion zone, and the volume content of the fillers in the polymer matrix was made to solve the problem. The high uniformity of the IR and UV pigments containing microcapsules over the PETG-filament functional layer planar distribution (Figure 8A) allowed the reversible color changing for the developed hybrid material. And it is controllable by the nature and the strength of environmental factors—heating and/or UV radiation.

**Figure 9:**
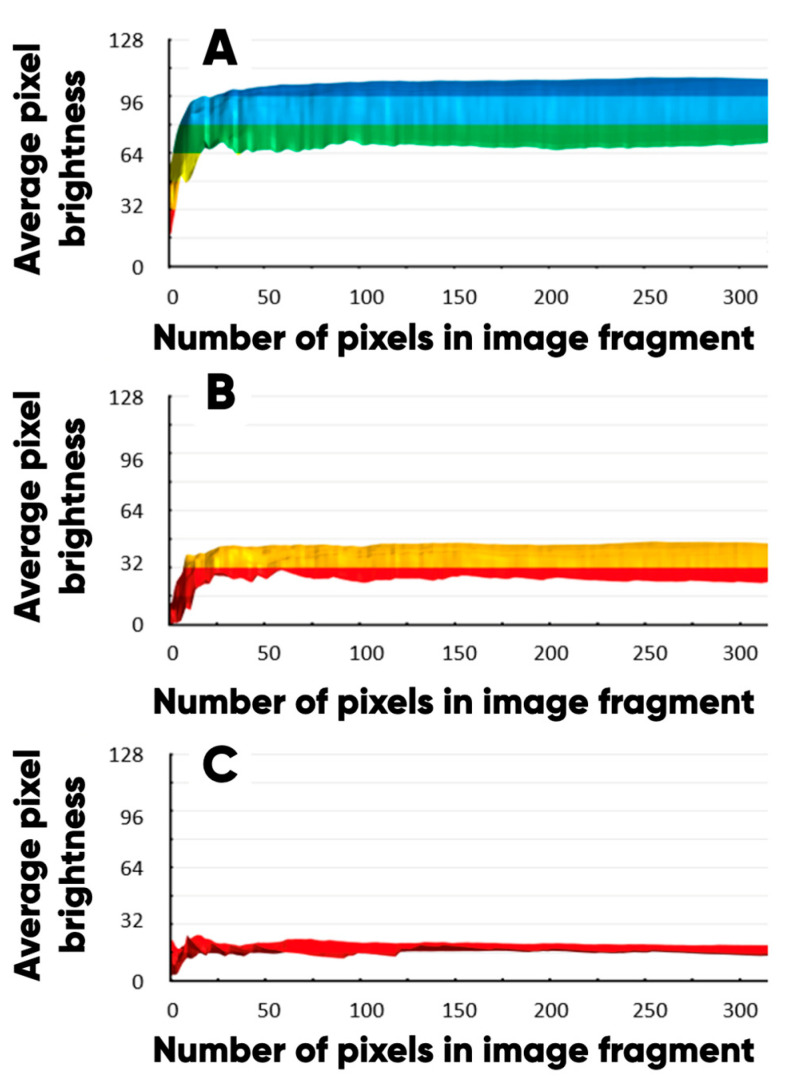
The dependence of the average pixel brightness of the carbon (**A**), oxygen (**B**), and nitrogen (**C**) planar distributions image fragments on the number of elements in the local data set.

The uniformity of the microcapsule distribution over the sample surface was quantified by plotting the dependence of the average pixel brightness values on the “northwest corner” technique [77,78,79,80], constructed data set size. The average pixel brightness level is a measure of the sample surface chemo-morphological heterogeneity with regard to a concrete chemical element. The color scale is used in Figure 9 to visualize the pixel average brightness intervals. The data set is representative when the average pixel brightness reaches a maximum level.

**Figure 10:**
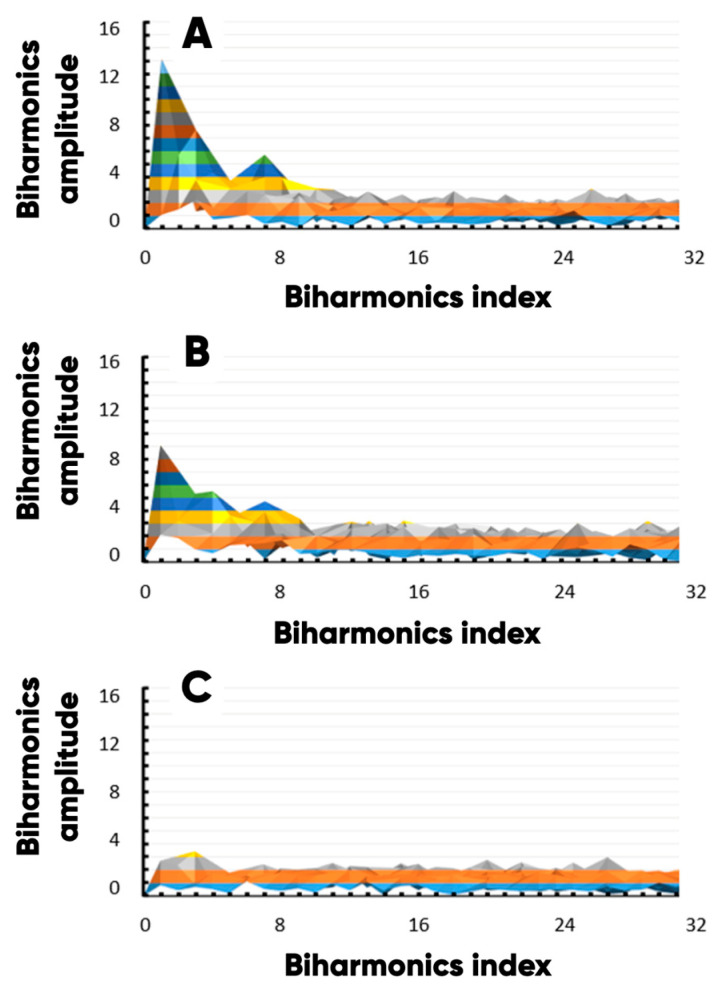
The carbon (**A**), oxygen (**B**), and nitrogen (**C**) planar distributions’ morphological spectra profile projections.

The highest values of the average pixel brightness were achieved for the carbon (Figure 9A); the lowest one (Figure 9C) was achieved for the nitrogen (Figure 8, N). The nitrogen in the surface layer of bulk modified PETG-filament is observed due to its presence in the IR- and UV-pigment microcapsules. The high uniformity of the microcapsules’ planar distribution is evidenced by a significantly smaller range of variation in the content of elemental nitrogen than oxygen and carbon (Figure 8A). Additionally, a comparative analysis of the functional layer’s surface chemo-morphological structure was carried out using the original technique [81,82,83] (Figure 10). The biharmonics amplitudes and indices are plotted along the vertical and the horizontal axis, respectively.

The first biharmonics amplitudes (characterizing the well-observed inhomogeneities of the planar distribution of the material-forming elements) are related as C:O:N = 12:9:3 (Figure 10). The observed unevenness in the nitrogen distribution is 4 times less than that of carbon and 3 times less than that of oxygen. Thus, the UV- or IR-induced luminescence intensity standard deviations are minimal due to the nitrogen-containing microcapsules are distributed over the sample’s surface evenly. Therefore, the product’s dye (microcapsules) containing the areas’ color is the same. The corresponding “statistical” error is not perceived by humans without specialized measuring equipment using [84,85]. So, high uniformity of the microcapsules’ planar distribution allows us to control the NWF/PETG-hybrid material color characteristics under the influence of the UV and/or IR radiation.

### 3.3. The Results of the NWF/PETG-Hybrid Material-Made Products Radio-Frequency Radiation Absorption Determining

**Figure 11:**
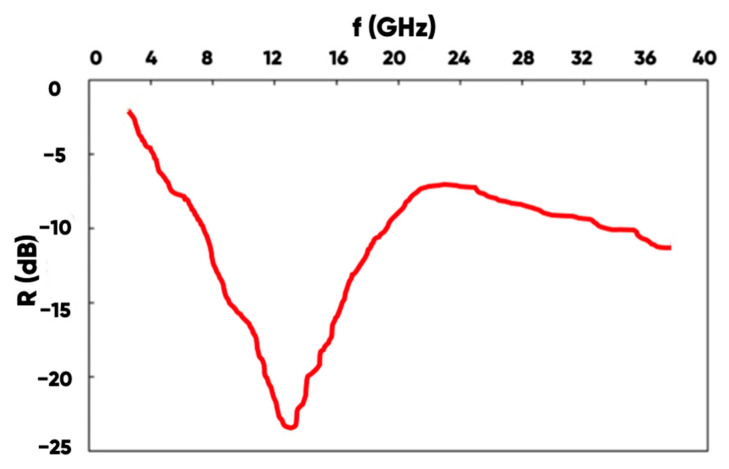
The radio-frequency range electromagnetic waves reflection coefficient spectrum for the NWF/PETG-hybrid material-made product (without aluminum foil rhombs periodic system).

The type of the obtained reflection coefficient on the electromagnetic waves frequency dependence (Figure 11) indicates the interference mechanism dominance in radio-frequency radiation absorption by the manufactured NWF/PETG-hybrid material [86].

**Figure 12:**
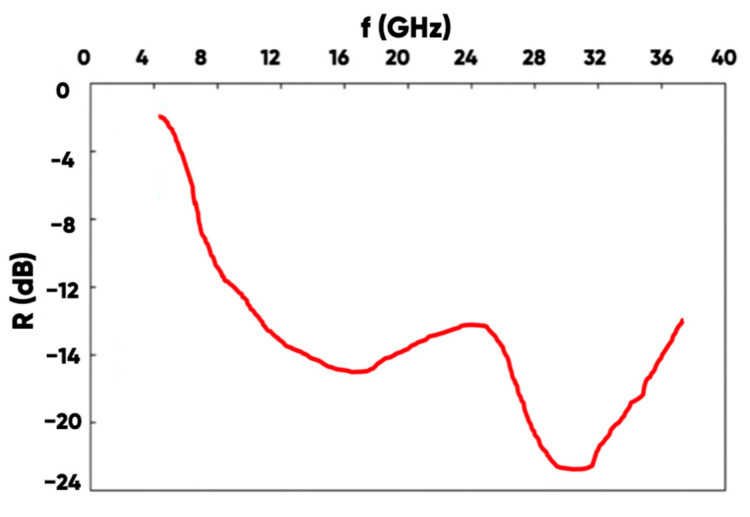
The radio-frequency range electromagnetic waves reflection coefficient spectrum for the NWF/PETG-hybrid material-made product with periodic aluminum foil rhombs system added.

A typical NWF/PETG sample has a minimum reflection coefficient (R = −23.0 dB) at a resonant frequency of f = 13.0 GHz. Moreover, the R-values do not exceed the target level of −10 dB in a fairly narrow range of 7.5–19.0 GHz. The spectrum of the radio-frequency electromagnetic radiation reflection coefficient for the hybrid material-made samples with the surface-added aluminum rhombs system is shown in Figure 12.

It can be seen (Figure 12) that the additional application of a 2D-periodic aluminum foil rhombs system provides an increase in radio-frequency electromagnetic field absorption in the extended (compared with the original) frequency range (from 7.5 to 37.5 GHz). It was found that the NWF modification (carried out by forming a functional layer from a UV-radiation cured resin (ABS-like Resin Pro 2, Anycubic) containing up to 5 vol.% of the carbon fibers) by photopolymer 3D-printing technique and the application of a 2D periodic system of the electrically conductive rhombs (with small and large diagonal sizes of ~5 and ~15 mm; with a distance between the elements of ~10 mm) provide an extension of the effective (at the level of 10.0 dB) electromagnetic field absorption into the short-wave range—from 19.0 (the extreme limit of the frequency range for the NWF/PETG sample) to 37.5 GHz.

It was found that the NWF modification (carried out by forming a functional layer from a UV-radiation cured resin (ABS-like Resin Pro 2, Anycubic) containing up to 5 vol. % of the carbon fibers) by the photopolymer 3D-printing technique and the application of a 2D periodic system of the electrically conductive rhombs (with small and large diagonal sizes of ~5 and ~15 mm; with a distance between the elements of ~10 mm) provide an extension of the effective (at the level of 10.0 dB) electromagnetic field absorption into the short-wave range—from 19.0 (the extreme limit of the frequency range for the NWF/PETG sample) to 37.5 GHz.

## 4. Conclusions

The main results of the paper are the technology of a hybrid electromagnetic radiation-absorbing material manufacturing and its detailed stages description. The obtained materials effectively absorb the electromagnetic field in the frequency range of 7.5 to 19 GHz with the non-woven felt (NWF) impregnated by the 5 vol.% carbon fibers containing UV-curable resin. And this range was expanded to 7.5 to 37.5 GHz by the 2D-periodic system of adding aluminum foil rhombs onto the NWF’s surface.

The contributions are that the electromagnetic fields’ absorption frequency range can be expanded by forming multilayer polymer composite material systems of various chemical and physical structures using the presented combination of bulk dispersed filling, gas-phase modification, and additive prototyping techniques.

The novelty is due to the innovative hybrid material-made prototype (consisting of the NWF basis, the IR-and UV-pigments bulk-modified PETG-filament functional layer, and the pattern of the glued electrically conductive elements (rhombs)) was created. The preliminary NWFs liquid-phase bulk modification was realized using the photo-curable 3D-printing technology. The PETG filament’s thermo- and photochromic microcapsules dispersed filling made it possible to achieve the effect of a reversible change in the color characteristics of the product under the influence of IR and/or UV radiation.

The direct applications of the developed materials can be carried out by shielding the research laboratories from the effects of external electromagnetic fields, ensuring the smooth functioning of high-tech industries, and achieving electromagnetic compatibility of a large number of electro-technical devices concentrated in small volumes of space.

It was shown that the combination of a rational choice of the NWF-base manufacturing technology, its impregnation with the carbon fiber-containing UV-curable resin, the formation of UV and IR pigments, bulk-modified PETG-filament 3D-printed layer, and electrically conductive elements added onto the hybrid material’s surface allows us to create multifunctional products with electromagnetic properties that can be directly regulated in radio- and optical-frequency ranges. In the future, we plan to explore the possibility of forming periodic systems of electrically conductive elements using 3D-printing techniques from various carbon nanotube bulk-modified filaments.

## Figures and Tables

**Table 1:** The results of the parameters specification for the model approximating the dependence of the effective strength modulus on the temperature and the speed of thermomechanical NWFs’ treatment.

Effective Strength Modulus E, MPa	Parameters of Approximating Model (1)				
	A, MPa	$T_{0},\;K$	δT, K	δv, m/min.	$R^{2},\;u\mathrm{n.}$
Longitudinal	58 ± 6	480 ± 50	25 ± 3	3.0 ± 0.3	0.89 ± 0.09
Transverse	47 ± 5	500 ± 50	37 ± 4	7.0 ± 0.7	0.89 ± 0.09

**Table 2:** The results of the tensile strength measurements for the NWF- (calendered at a speed of 6 m/min at different temperatures) and based on the NWF/PETG hybrids.

Sample Type	Ultimate Tensile Strength σ, MPa						
	Calendar Rolling Temperature, °C						
	20	110	130	150	170	190	210
NWF	6.0 ± 0.6	5.7 ± 0.6	2.9 ± 0.3	3.1 ± 0.3	4.4 ± 0.5	5.7 ± 0.6	4.8 ± 0.5
NWF/PETG	8.1 ± 0.8	18 ± 2	11 ± 1	8.1 ± 0.8	8.3 ± 0.8	12 ± 1	6.1 ± 0.6

## Data Availability

Data are contained within the article.
